# Comparison of Resilience Among Healthcare Workers During the COVID-19 Pandemics: A Multinational Cross-Sectional Survey in Southeast Asian Jurisdictions

**DOI:** 10.3389/ijph.2022.1605505

**Published:** 2022-12-21

**Authors:** Eliza Lai-yi Wong, Hong Qiu, Wai Tong Chien, Cho Lee Wong, Hom Nath Chalise, Huong Thi Xuan Hoang, Hong Trang Nguyen, Shu-Fang Wang, Jian Tao Lee, Yu-Nu Chen, Paul Kay-sheung Chan, Martin Chi-sang Wong, Annie Wai-ling Cheung, Eng-kiong Yeoh

**Affiliations:** ^1^ JC School of Public Health and Primary Care, Faculty of Medicine, The Chinese University of Hong Kong, Hong Kong, Hong Kong SAR, China; ^2^ Centre for Health Systems and Policy Research, JC School of Public Health and Primary Care, Faculty of Medicine, The Chinese University of Hong Kong, Hong Kong, Hong Kong SAR, China; ^3^ The Nethersole School of Nursing, Faculty of Medicine, The Chinese University of Hong Kong, Hong Kong, Hong Kong SAR, China; ^4^ Department of Public Health, Nobel College, Affiliated to Pokhara University, Kathmandu, Nepal; ^5^ Faculty of Nursing, Phenikaa University, Hanoi, Vietnam; ^6^ Department of Nursing, Tzu Chi University of Science and Technology, Hualien city, Taiwan; ^7^ School of Nursing, College of Medicine, Chang Gung University, Tao-Yuan, Taiwan; ^8^ Nursing Department, Chang Gung Memorial Hospital, Linkou Branch, Tao-Yuan, Taiwan; ^9^ Department of Microbiology, Faculty of Medicine, The Chinese University of Hong Kong, Hong Kong, Hong Kong SAR, China; ^10^ Stanley Ho Centre for Emerging Infectious Diseases, JC School of Public Health and Primary Care, Faculty of Medicine, The Chinese University of Hong Kong, Hong Kong, Hong Kong SAR, China; ^11^ Centre for Health Education and Health Promotion, JC School of Public Health and Primary Care, Faculty of Medicine, The Chinese University of Hong Kong, Hong Kong, Hong Kong SAR, China

**Keywords:** healthcare workers, resilience, organizational support, Southeast Asia, workplace policy

## Abstract

**Objectives:** To examine the level of resilience among the frontline healthcare workers (HCWs) in four different Southeast Asian jurisdictions and identify the potential factors that may enhance healthcare workers resilience.

**Methods:** An online cross-sectional survey was carried out among 3,048 eligible healthcare workers in Hong Kong, Nepal, Vietnam, and Taiwan from May 2021 to July 2022, and information on individual resilience, socio-demographic characteristics, organizational supports, and personal exposures were collected. A binary logistic regression model was used to identify the factors that were associated with a high resilience level.

**Results:** The resilience score was the highest among healthcare workers of Vietnam, followed by Taiwan and Hong Kong, with Nepal scoring the lowest. Participants with old age, part-time work, higher education level, more satisfaction with workplace policy, better organizational supports, and fewer COVID-specific worries were associated with higher resilience. Healthcare workers who were satisfied with the overall organizational policy support had an OR of 1.48 (95% CI: 1.25–1.76) for a high resilience level.

**Conclusion:** Implementing satisfying organizational policies and establishing supportive work environments for frontline healthcare workers can increase individual resilience and organizational stability.

## Introduction

Resilience refers to the ability to successfully and positively adapt to stressful or traumatic events, and thus has a protective effect on mental health following exposure to such events [1]. Operating on the frontline and having to deal with more than 2 years of the COVID-19 pandemic since 2020, healthcare workers (HCWs) have presented more mental health disorders such as the symptoms of anxiety, depression, as well as low resilience [2]. An integrative review of studies on resilience during the COVID-19 pandemic, revealed that building resilience in HCWs can serve as a protective factor on distress experienced, anxiety and depression as well as against negative outcomes related to the job, including burnout [2–5]. Moreover, a higher resilience level was found to be associated with positive outcomes related to the job, including a higher intention to receive a COVID-19 vaccination, reinforcing posttraumatic growth, and lower burnout [6, 7]. Resilience has also been demonstrated to act as a mediator for wellbeing and healthcare system performance [8, 9].

Managing the pandemic has tested the capacity of the healthcare system, by stretching available resources to cope with the demands levelled by COVID-19. In early 2022, the World Health Organization (WHO) recommended building resilient health systems to support countries recovering from the pandemic [10], and resilient healthcare supply chains to manage pandemics in low- and middle-income countries [11]. Despite shared pandemic challenges, individual jurisdictions have initiated different organizational policies and measures to build resilience; however, the resilience level of the frontline HCWs against the backdrop of the broader context and multidimensional policy response, have not been studied.

Southeast Asian jurisdictions were among the first to be exposed to and affected by COVID-19. To increase HCWs’ resilience, different policies or initiatives were introduced. For example, Hong Kong (HK), Nepal, Taiwan and Vietnam adopted an approach combining contact tracing, isolation and quarantine for infected as well as close contact cases, to mitigate the pandemic risk. Also, hospitals provided regular communication through either online or social media platforms to update organizational policies and measures to all staff. To further relieve the workload, they allocated HCWs from other specialties and healthcare professionals to take care of COVID cases. Hospitals in Vietnam also provided telehealth consultation and monitoring *via* phone for COVID cases that do not require hospitalization. Frontline HCWs are one of the priority groups eligible for free vaccination programs, and duty allowance has been provided for HCWs taking care of COVID cases in all four jurisdictions. In addition, HK and Taiwan provided accommodation allowances, while Taiwan and Vietnam provided further financial support for living, such as electricity for those HCWs working in social welfare during duty. Details of policies and initiatives in the four jurisdictions are shown in Table 1.

**TABLE 1:** Contextual environments in four jurisdictions (Southeast Asia, 2021–2022).

Jurisdiction	Hong Kong	Nepal	Vietnam	Taiwan
Context				
Experience of SARS cases between 1 Nov 2002 and 31 July 2003 ^a^	1755 incidence cases and 299 deaths	No	63 incidence cases and 5 deaths	346 incidence cases and 37 deaths
Covid pandemic during the survey period ^b^				
Time of Survey	4th wave	3rd wave	4th wave	4th wave
	11 May—23 June 2021	10 August—7 November 2021	12 July—20 November 2021	14 December 2021–30 July 2022
Averaged Daily Incident Rate per 100,000 population	0.03	3.59	8.19	83.11
Case fatality Rate	0.81%	1.37%	2.24%	0.18%
Organization strategies during COVID pandemic up to the survey period ^c-h^				
Approach	Control the potential waves with a combination of contact tracing, isolation, and quarantine	Control the potential waves with a combination of contact tracing, isolation, and quarantine	Control the potential waves with a combination of contact tracing, quarantine, and 5K policy (mandatory face mask, cleaning and disinfection (hands and high-touch surfaces), no gathering, keeping a safe distance from others, and health declaration)	Control the potential waves with a combination of contact tracing, reinforced border quarantine, cross-border collaboration, community quarantine, medical care institution, inspection policy, international exchange, and applying video consultation
Organization Communication	Regular online communication forum to update organization policy for staff	Regular communication forum (such as viber and email group) to update organization policy for staff	Regular online communication forum to update organization policy for staff	Daily live communication and broadcasts on television by The Central Epidemic Command Center for updating the government policies for HCWs and people
Assignment of Duty	Assigned HCWs from other specialties including allied healthcare professionals to take care of COVID cases	Assigned HCWs including fresh one as well to take care of COVID cases	Assigned HCWs from other specialties, retired HCWs, and medical/nursing students (final year) to take care of COVID cases in the hospital	Assigned HCWs from other specialties (medical care institutions, nursing care institutions, and social welfare agencies) to take care of COVID cases
			Provide telehealth consultation and monitoring *via* phone for COVID cases that are not required hospitalization	
			Hospitals in low COVID cases provinces send their HCWs to support HCWs in COVID hotspots province (mostly in the South of Vietnam including Ho Chi Minh City, Binh Duong, ect)	
Benefits Package	Priority of the use of vaccines for HCW	Priority of the use of vaccines for HCW	Priority of the use of vaccines for HCWs and their families	Prioritization of vaccine use for HCW
	Duty allowance provided for HCWs taking care of COVID cases	Duty allowance provided for HCWs taking care of COVID cases	Duty allowances provided for HCWs taking care of COVID cases	Duty allowances provided for HCWs and non HCWs taking care of COVID cases
	Accommodation allowance provided for HCWs taking care of COVID cases		Accommodation and meals for HCWs taking care of COVID cases	Accommodation allowances or related vouchers for HCWs taking care of COVID cases
				Electricity fee waiver for social welfare agencies and healthcare institutions

^a^ Summary of probable SARS cases with onset of illness from 1 November 2002 to 31 July 2003 (Based on data as of 31 December 2003.). World Health Organization. Accessed on 10 August 2022. https://www.who.int/publications/m/item/summary-of-probable-sars-cases-with-onset-of-illness-from-1-november-2002-to-31-july-2003.

^b^ Data on COVID-19 (coronavirus). Our World in Data, Published 2022. Accessed 3 August 2022. https://github.com/owid/covid-19-data/tree/master/public/data.

^c^ Together, we fight the virus. The Government of the Hong Kong Special Administrative Region. Accessed 10 August 2022. https://www.coronavirus.gov.hk/eng/index.html.

^d^ Health sector policy responses and health workforce management during COVID-19 in Nepal: Lessons for building resilient health systems—a policy brief. Accessed 9 August 2022. https://www.herdint.com/wp-content/uploads/2022/05/Nepal-May-2022.pdf.

^e^ Crucial policies for combating COVID-19. Ministry of Health and Welfare of Taiwan. Accessed 10 August 2022. https://covid19.mohw.gov.tw/en/sp-timeline0-206.html.

^f^ Taiwan Centers for Disease Control. Prevention and Control of COVID-19 in Taiwan. Accessed 3 August 2022. https://www.cdc.gov.tw/En/Category/Page/0vq8rsAob_9HCi5GQ5jH1Q.

^g^ The Ministry of Health recommends “5K” to live safely with the epidemic. Accessed 10 August 2022. https://covid19.gov.vn/bo-y-te-khuyen-cao-5k-chung-song-an-toan-voi-dich-benh-1717130215.htm.

^h^ Ministry of Health Portal. OVERVIEW: Seminar on 'COVID-19 pandemic and policies for healthcare workers’. Accessed 9 August 2022. https://moh.gov.vn/home.

As the severe acute respiratory syndrome coronavirus 2 (SARS-CoV-2) may become an endemic virus in the future, building the resilience of frontline HCWs should be an essential strategy for posttraumatic growth at both individual and organizational levels, to face new COVID variants more confidently as they emerge over time. In the current study, we aimed to examine the level of resilience of frontline HCWs in four Southeast Asian jurisdictions, including the high-income regions of HK and Taiwan, as well as middle-income countries such as Nepal and Vietnam, and identify the potential associated factors. Findings from this study may provide useful information to build resilience among HCWs.

## Methods

### Study Setting and Participants

This cross-sectional study was carried out in four Southeast Asian jurisdictions—namely HK, Taiwan, Nepal and Vietnam—through an online self-administered questionnaire. The survey was conducted with frontline nurses in Hong Kong from 11 May to 23 June; on HCWs (nurses and doctors) in Nepal from 10 August to 7 November; Vietnam from 12 July to 20 November in the year 2021; and Taiwan from 14 December 2021 to 30 July 2022. We disseminated the e-mail invitations to Hong Kong nurses to participate in this study through the Association of Hong Kong Nursing Staff. For Vietnam, Nepal, and Taiwan, the invitation letters and the online questionnaire link or QR code were made available to the target population through email and multiple social networks. We used the online survey platform “Qualtrics” to create the questionnaire, distribute, and store the collected responses, which was subscribed to by The Chinese University of Hong Kong. Eligible study participants were nurses or doctors aged 18 or above, working in either public or private healthcare settings, and working on a full-time or part-time basis. Informed consent was obtained from each respondent online before taking the survey. The study was approved by the Joint Chinese University of Hong Kong—New Territories East Cluster Clinical Research Ethics Committee. All procedures performed in the current study involving human participants were in accordance with the ethical standards of the institutional research committee and with the 1964 Helsinki declaration and its later amendments or comparable ethical standards.

### Data Collection and Measures

The structured questionnaire was developed based on previous international and local studies [12, 13]. Information about resilience, organizational support, socio-demographic characteristics (such as age group, gender, work type, job type, and education level), and personal exposures were collected.

*Resilience* referred to a stress-coping ability and could potentially buffer the adverse effects of traumatic events to protect against mental illnesses, as measured by the Connor–Davidson Resilience Scale (CD-RISC) [14]. An abbreviated form of the scale consisting of two items (CDRISC2)—“Able to adapt when changes occur” and “Tend to bounce back after illness, injury, or other hardships”—using a 5-point Likert-type response scale from not true at all (0) to true nearly all the time (4), was employed. Total score ranged from 0 to 8, with a higher score corresponding to a higher level of resilience. The CD-RISC2 has been demonstrated to have good test-retest reliability, convergent and discriminant validity as well as significant correlation with the overall CD-RISC score [14]; and the Chinese version of the CD-RISC2 has been shown as a reliable and valid measure of resilience assessment in the Hong Kong population [15]. The overall internal consistency of the CD-RISC2 was good (the Cronbach’s alpha = 0.764 [95% CI: 0.747–0.781]) in the current study.

*Organizational support: Attitudes towards organizational policy* referred to the views on workplace infection control and prevention policy and guideline in terms of comprehensiveness, clarity, timely, transparency, and effectiveness, using a 5-point scale from extremely unsatisfied (1) to extremely satisfied (5). We calculated the frequency and proportion of those satisfied with the guideline and identified the organizational support of the overall infection control and prevention guideline, as either comprehensive, clear, timely, transparent, or effective. *Company required regular virus testing* was also considered as organizational support. *COVID-specific worries* were perceived risks of protective equipment shortage at the workplace and infecting family members due to the work, using a 5-point scale from not worried at all (1) to extremely worried (5). Fewer worries meant better organizational support.

*Personal exposures* were measured through the questions including self-reported long-term consultation and regular medication, self or someone known ever been diagnosed with COVID-19 and ever been under compulsory quarantine, and number of patients encountered with suspected or confirmed COVID-19 infection.

### Statistical Analysis

Descriptive analyses were performed to summarize the general information, personal exposures, workplace policy, organizational support, and COVID-specific worries in the study samples. The Chi-square test for categorical variables and non-parametric Kruskal Wallis test for continuous variables, were used to compare such characteristics among the four jurisdictions. A bar chart with error bar was used to present the mean and standard deviation (SD) of CD-RISC2 score for each city. Spearman correlation coefficient matrix was then presented to show the correlation among resilience score and main study variables.

We used median of CD-RISC2 score as a cutoff point to identify the high (>4) and low (≤4) resilience groups and the associations of socio-demographic factors, personal exposures, workplace policy, organizational support, and COVID-specific worries with high resilience group using binary logistic regression. First, univariate logistic regressions were performed to derive the crude odds ratio (OR) and corresponding 95% confidence interval (CI), to present the potential risk of each variable of interest on resilience. Second, we included all the variables of interest into the model and conducted a backward stepwise multivariate model [6], to identify the most significant associated factors after adjusting confounding factors from each other. Data processing and all analyses were conducted using R version 4.1.2 (R Foundation for Statistical Computing, Vienna, Austria). Any *p* < 0.05 was regarded as statistically significant.

## Results

### Demographics and Personal Exposures

Among the 3,048 eligible HCWs who participated in the survey, 2,176 were from Hong Kong, 328 were from Nepal, 145 were from Vietnam, and 399 were from Taiwan. The social-demographic characteristics and personal exposures of the participants in the four jurisdictions are compared in Table 2. The proportion of participants younger than 40 years of age among the participants from Vietnam was 81.4%, significantly higher than that in Nepal (58.3%), Taiwan (52.9%) and HK (48.9%). Female participants were dominant at 88.0% in HK, 78.2% in Taiwan, 69.0% in Vietnam, and 64.3% in Nepal. 87.1% of the participants were full-time employees, 89.9% of them were nurses, and 79.6% of them had a bachelor’s degree or above. The proportion on self-reported long-term consultation and regular medication was highest in Vietnam (70.3%), followed by 50.6% in Nepal, 25.9% in Hong Kong, and the lowest in Taiwan (20.6%). Participants in Nepal had a significantly higher proportion of self or someone known who had been diagnosed with COVID-19 (44.5% and 91.5%, respectively), and higher proportion of ‘ever been under compulsory quarantine’ (74.1%), compared with those in Hong Kong, Taiwan and Vietnam (Table 2). On average, the HCWs encountered four patients every day, among which an average of two were suspected or confirmed COVID-19 cases.

**TABLE 2:** The general characteristics and personal exposure of healthcare workers during the COVID-19 pandemics in four jurisdictions (Southeast Asia, 2021–2022)^a^.

Characteristics	Total (N = 3048)	Hong Kong (N = 2176)	Nepal (N = 328)	Vietnam (N = 145)	Taiwan (N = 399)
General information, N (%)					
Age group					
18–29 years	577 (18.9)	353 (16.2)	97 (29.6)	46 (31.7)	81 (20.3)
30–39 years	1007 (33.0)	711 (32.7)	94 (28.7)	72 (49.7)	130 (32.6)
40–49 years	760 (24.9)	547 (25.1)	68 (20.7)	23 (15.9)	122 (30.6)
≥50 years	704 (23.1)	565 (26.0)	69 (21.0)	4 (2.8)	66 (16.5)
Sex^b^					
Male	506 (16.6)	257 (11.8)	117 (35.7)	45 (31.0)	87 (21.8)
Female	2538 (83.3)	1915 (88.0)	211 (64.3)	100 (69.0)	312 (78.2)
Work type					
Full-time	2656 (87.1)	1912 (87.9)	246 (75.0)	119 (82.1)	379 (95.0)
Part-time	392 (12.9)	264 (12.1)	82 (25.0)	26 (17.9)	20 (5.0)
Job type					
Nurse	2741 (89.9)	2176 (100.0)	193 (58.8)	105 (72.4)	267 (66.9)
Doctor	307 (10.1)	0	135 (41.2)	40 (27.6)	132 (33.1)
Education					
Diploma or below	623 (20.4)	536 (24.6)	34 (10.4)	34 (23.4)	19 (4.8)
Bachelor’s degree	1426 (46.8)	964 (44.3)	114 (34.8)	68 (46.9)	280 (70.2)
Master’s or doctoral degree	999 (32.8)	676 (31.1)	180 (54.9)	43 (29.7)	100 (25.1)
Personal exposure, N (%)					
Self-reported long-term follow-up consultation and regular medication	913 (30.0)	563 (25.9)	166 (50.6)	102 (70.3)	82 (20.6)
Ever been diagnosed with COVID-19	238 (7.8)	26 (1.2)	146 (44.5)	2 (1.4)	64 (16.0)
Someone known diagnosed with COVID-19	1031 (33.8)	419 (19.3)	300 (91.5)	23 (15.9)	289 (72.4)
Ever been under compulsory quarantine	472 (15.5)	76 (3.5)	243 (74.1)	54 (37.2)	99 (24.8)
No. of patients encountered every day (Mean ± SD)	4.3 ± 1.9	4.3 ± 2.0	3.8 ± 0.9	4.4 ± 2.2	4.4 ± 1.9
No. of patients with suspected/confirmed COVID-19 infection encountered (Mean ± SD)	2.3 ± 1.6	2.0 ± 1.6	2.8 ± 1.0	2.7 ± 2.2	3.1 ± 2.0

^a^ The *p*-values for overall comparison among the four regions got from the Chi-square test for categorical variables and non-parametric Kruskal Wallis test for continuous variables were all <0.05.

^b^ There were four missing values in sex in Hong Kong.

### Resilience

**FIGURE 1:**
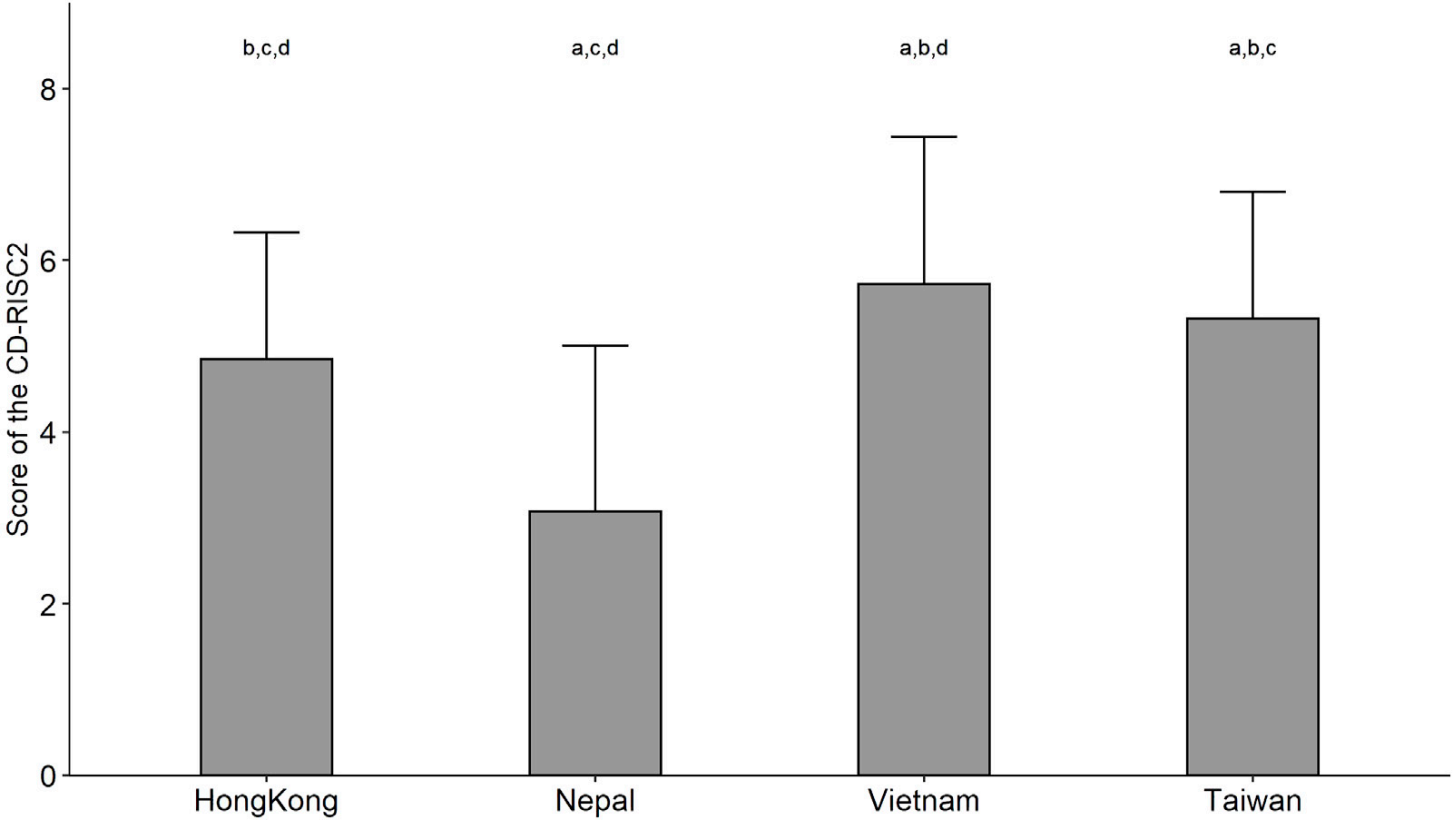
The score of the 2-item Connor–Davidson Resilience Scale among healthcare workers in four jurisdictions (Southeast Asia, 2021–2022). Notes: The grey bar shows the mean and the error bar shows the standard deviation of the score. Wilcoxon rank sum test with Bonferroni correction was used for pairwise comparisons among the four jurisdictions. a: compared with Hong Kong, *p* < 0.01; b: compared with Nepal, *p* < 0.01; c: compared with Vietnam, *p* < 0.01; d: compared with Taiwan, *p* < 0.01.

The score of CD-RISC2 ranged from 0 to 8, with a mean score of 5.7 for the participants from Vietnam, 5.3 for Taiwan, 4.8 for Hong Kong and 3.1 for Nepal—showing the highest level of resilience in the HCWs from Vietnam, followed by Taiwan and Hong Kong, and the lowest in Nepal (Figure 1 and Table 3). Spearman correlation matrix among resilience score and the major variables, showed the correlation coefficients were low to moderate (data not shown).

**TABLE 3:** The resilience and organizational support for healthcare workers during the COIVD-19 pandemics in four jurisdictions (Southeast Asia, 2021–2022)^a^.

Variables	Total (N = 3048)	Hong Kong (N = 2176)	Nepal (N = 328)	Vietnam (N = 145)	Taiwan (N = 399)
Resilience					
Item1: I am able to adapt when change occurs. N (%)					
Not true at all (0)	126 (4.1)	45 (2.1)	68 (20.7)	4 (2.8)	9 (2.3)
Rarely true (1)	290 (9.5)	161 (7.4)	106 (32.3)	3 (2.1)	20 (5.0)
Sometimes true (2)	1116 (36.6)	864 (39.7)	85 (25.9)	32 (22.1)	135 (33.8)
Often true (3)	1259 (41.3)	970 (44.6)	56 (17.1)	57 (39.3)	176 (44.1)
True nearly all the time (4)	257 (8.4)	136 (6.2)	13 (4.0)	49 (33.8)	59 (14.8)
Item2: I tend to bounce back after illness, injury, or other hardships. N (%)					
Not true at all (0)	137 (4.5)	45 (2.1)	80 (24.4)	8 (5.5)	4 (1.0)
Rarely true (1)	313 (10.3)	193 (8.9)	94 (28.7)	6 (4.1)	20 (5.0)
Sometimes true (2)	1175 (38.5)	935 (43.0)	63 (19.2)	42 (29.0)	135 (33.8)
Often true (3)	1170 (38.4)	869 (39.9)	72 (22.0)	50 (34.5)	179 (44.9)
True nearly all the time (4)	253 (8.3)	134 (6.2)	19 (5.8)	39 (26.9)	61 (15.3)
Total score of CD-RISC2^b^ (Mean ± SD)	4.8 ± 1.7	4.8 ± 1.5	3.1 ± 1.9	5.7 ± 1.7	5.3 ± 1.5
Low resilience (CD-RISC2 ≤ 4)	1344 (44.1)	947 (43.5)	243 (74.1)	34 (23.4)	120 (30.1)
High resilience (CD-RISC2 > 4)	1704 (55.9)	1229 (56.5)	85 (25.9)	111 (76.6)	279 (69.9)
Organizational support. N (%)					
Overall infection control and prevention guideline					
Comprehensiveness	1645 (54.0)	1140 (52.4)	133 (40.5)	115 (79.3)	257 (64.4)
Clarity	1672 (54.9)	1165 (53.5)	133 (40.5)	116 (80.0)	258 (64.7)
Timely	1525 (50.0)	1026 (47.2)	136 (41.5)	113 (77.9)	250 (62.7)
Transparency	1565 (51.3)	1060 (48.7)	138 (42.1)	119 (82.1)	248 (62.2)
Effectiveness	1522 (49.9)	1015 (46.6)	147 (44.8)	109 (75.2)	251 (62.9)
Organizational policy support ^c^	2082 (68.3)	1382 (63.5)	267 (81.4)	130 (89.7)	303 (75.9)
Company ever required employees to undergo regular virus testing	2221 (72.9)	1601 (73.6)	263 (80.2)	110 (75.9)	247 (61.9)
Worried about workplace supply of protective equipment					
Not worried at all	415 (13.6)	281 (12.9)	35 (10.7)	38 (26.2)	61 (15.3)
Slightly worried	1361 (44.7)	1090 (50.1)	37 (11.3)	64 (44.1)	170 (42.6)
Moderately worried	839 (27.5)	605 (27.8)	94 (28.7)	26 (17.9)	114 (28.6)
Severe worried	328 (10.8)	148 (6.8)	127 (38.7)	13 (9.0)	40 (10.0)
Extremely worried	105 (3.4)	52 (2.4)	35 (10.7)	4 (2.8)	14 (3.5)
Worried about infecting family members					
Not worried at all	103 (3.4)	80 (3.7)	8 (2.4)	9 (6.2)	6 (1.5)
Slightly worried	822 (27.0)	681 (31.3)	20 (6.1)	64 (44.1)	57 (14.3)
Moderately worried	1362 (44.7)	1087 (50.0)	98 (29.9)	28 (19.3)	149 (37.3)
Severe worried	492 (16.1)	208 (9.6)	155 (47.3)	33 (22.8)	96 (24.1)
Extremely worried	269 (8.8)	120 (5.5)	47 (14.3)	11 (7.6)	91 (22.8)

^a^ The *p*-values for overall comparison among the four regions got from the Chi-square test for categorical variables and non-parametric Kruskal Wallis test for continuous variables were all <0.05.

^b^ The 2-item Connor–Davidson Resilience Scale (CD-RISC2) is a 2-item scale using a 5-point Likert-type response scale from not true at all (0) to true nearly all of the time (4). Total scores range from 0 to 8, with higher scores corresponding to higher levels of resilience.

^c^ Organizational policy support was identified that the overall infection control and prevention guideline was either comprehensive, clear, timely, transparent, or effective.

### Organizational Support

Participants from Vietnam had significantly higher proportion of satisfaction with the comprehensiveness, clarity, timeliness, transparency, and effectiveness of the workplace infection control and prevention guideline (75.2%–82.1%) than those of Taiwan (62.2%–64.7%), Hong Kong (46.6%–53.5%) and Nepal (40.5%–44.8%). 61.9%–80.2% participants reported that their company had ever required employees to undergo regular virus testing. More participants from Nepal worried about the shortage of workplace protective equipment and on infecting family members than Taiwan, Hong Kong and Vietnam (Table 3).

### Potential Factors Associated With Resilience

The potential factors associated with resilience among the HCWs in the four jurisdictions were examined in the univariate and multivariate logistic regression model, respectively (Table 4). Compared with Hong Kong nurses, the HCWs from Nepal exhibited a statistically significant lower level of resilience with an OR of 0.31 (95% CI: 0.23–0.42); while the HCWs from Vietnam and Taiwan had higher level of resilience with an OR of 2.93 (1.92–4.49) and 2.20 (1.70–2.85), respectively.

**TABLE 4:** Association of demographics, policy/organizational support and perceived risks with resilience among healthcare workers during the COVID-19 pandemic in four jurisdictions (Southeast Asia, 2021–2022)^a^.

Variables	Resilience level N (%)		Binary logistic regression [OR (95% CI)]	
	Low (CD-RISC2 ≤ 4) (N = 1344)	High (CD-RISC2 > 4) (N = 1704)	Univariate model	Backward stepwise multivariate model
Jurisdiction				
Hong Kong	947 (70.5)	1229 (72.1)	1.00	1.00
Nepal	243 (18.1)	85 (5.0)	**0.27 (0.21, 0.35)**	**0.31 (0.23, 0.42)**
Vietnam	34 (2.5)	111 (6.5)	**2.52 (1.70, 3.73)**	**2.93 (1.92, 4.49)**
Taiwan	120 (8.9)	279 (16.4)	**1.79 (1.42, 2.26)**	**2.20 (1.70, 2.85)**
General information				
Age group				
18–29 years	309 (23.0)	268 (15.7)	1.00	1.00
30–39 years	481 (35.8)	526 (30.9)	**1.26 (1.03, 1.55)**	1.08 (0.86, 1.35)
40–49 years	317 (23.6)	443 (26.0)	**1.61 (1.30, 2.00)**	**1.28 (1.01, 1.64)**
≥50 years	237 (17.6)	467 (27.4)	**2.27 (1.81, 2.85)**	**1.89 (1.46, 2.44)**
Sex^b^				
Male	233 (17.3)	273 (16.0)	1.00	—
Female	1109 (82.5)	1429 (83.9)	1.10 (0.91, 1.33)	—
Work type				
Full-time	1184 (88.1)	1472 (86.4)	1.00	1.00
Part-time	160 (11.9)	232 (13.6)	1.17 (0.94, 1.45)	**1.32 (1.03, 1.68)**
Job type				
Doctor	135 (10.0)	172 (10.1)	1.00	—
Nurse	1209 (90.0)	1532 (89.9)	0.99 (0.78, 1.26)	—
Education				
Diploma or below	322 (24.0)	301 (17.7)	1.00	1.00
Bachelor’s degree	629 (46.8)	797 (46.8)	**1.36 (1.12, 1.64)**	**1.32 (1.07, 1.61)**
Master’s or doctoral degree	393 (29.2)	606 (35.5)	**1.65 (1.35, 2.02)**	**1.88 (1.50, 2.35)**
Organizational support				
Overall infection control and prevention guidelines (Organizational policy support) ^c^	822 (61.2)	1260 (73.9)	**1.80 (1.54, 2.10)**	**1.48 (1.25, 1.76)**
Company ever required employees to undergo regular virus testing	964 (71.7)	1257 (73.8)	1.11 (0.94, 1.30)	**1.23 (1.03, 1.46)**
Worried about the workplace supply of protective equipment ^d^	2.7 ± 1.0	2.3 ± 0.9	**0.63 (0.58, 0.68)**	**0.80 (0.73, 0.88)**
Worried about infecting family members ^d^	3.2 ± 1.0	2.9 ± 0.9	**0.71 (0.66, 0.77)**	**0.81 (0.74, 0.89)**
Personal exposure				
Self-reported long-term follow-up consultation and regular medication	419 (31.2)	494 (29.0)	0.90 (0.77, 1.05)	**0.79 (0.66, 0.95)**
Ever been diagnosed with COVID-19	134 (10.0)	104 (6.1)	**0.59 (0.45, 0.77)**	—
Someone known diagnosed with COVID-19	498 (37.1)	533 (31.3)	**0.77 (0.66, 0.90)**	—
Ever been under compulsory quarantine	252 (18.8)	220 (12.9)	**0.64 (0.53, 0.78)**	—
No. of patients encountered every day (per 1 increase) ^d^	4.3 ± 1.8	4.2 ± 2.0	0.99 (0.95, 1.03)	—
No. of patients with suspected/confirmed COVID-19 infection encountered (per 1 increase) ^d^	2.2 ± 1.6	2.3 ± 1.8	1.01 (0.97, 1.05)	—

^a^ The resilience score was categorized by a cutoff point of four to identify the high (>4) and low (≤4) resilience groups. % by column was presented and OR (95% CI) was estimated from the binary logistic regression. OR in bold means statistically significant association.

^b^ Four missing values in sex have been excluded.

^c^ Organizational policy support was identified that the overall infection control and prevention guideline was either comprehensive, clear, timely, transparent, or effective.

^d^ Included in the model as a continuous variable.

Among the socio-demographic characteristics, HCWs with old age, part-time work type, and higher education level tended to have a higher level of resilience, when compared with the age group of 18–29 years, OR of the high resilience group was 1.28 (1.01–1.64) for the age of 40–49 years, and 1.89 (1.46–2.44) for the age ≥50 years. Part-time workers had higher resilience than full-time ones, with an OR of 1.32 (1.03–1.68). Compared with diploma or below level of education, bachelor’s degree and master’s/doctoral degree holders had an OR of 1.32 (1.08–1.62) and 1.87 (1.50–2.35), respectively.

Organizational support was associated with the high resilience, while a higher perceived risk (COVID-specific worries) was associated with a lower resilience. HCWs who were satisfied with the overall infection control and prevention guideline (either comprehensive, clear, timely, transparent, or effective) had an OR of 1.48 (1.25–1.76) and exhibited a high resilience level. Introducing a company requirement for regular virus testing, could increase the HCWs’ resilience with an OR of 1.23 (1.03–1.46). HCWs who were worried about the shortage of protective equipment in the workplace and infecting family members tended to have lower resilience (OR = 0.80–0.81).

Participants with self-reported long-term follow-up consultation and regular medication, were found to have a lower resilience score with an OR of 0.79 (0.66–0.95). Although the personal exposures (self or someone known ever been diagnosed with COVID-19, ever been under compulsory quarantine) were identified as lower in the resilience score in the univariate models, such ORs lost statistical significance in the stepwise multivariate model.

## Discussion

As the first survey to study HCWs’ resilience during the COVID-19 pandemic in the four Southeast Asia jurisdictions, we observed the highest resilience level in the HCWs of Vietnam, followed by Taiwan and Hong Kong, and the lowest resilience level in Nepal in responding to the broader context of multi-dimensional policy. Participants with old age, part-time work, higher education level, satisfaction with workplace policy, and organizational support were associated with a higher resilience; on the other hand, the COVID-specific worries (perceived risk) and self-reported chronic health conditions were likely attributed to a low resilience level.

We observed a slightly lower resilience score of 4.8 in Hong Kong nurses during the pandemic, compared with the mean CD-RISC2 score of 5.03 in the Hong Kong population pre-COVID-19 pandemic [15]. This was consistent with the findings from an integrative review of studies that resilience scores among frontline HCWs worldwide were in the moderate range during the COVID-19 pandemic; and epidemiological data from the United States and China also showed a decrease in nurse resilience when compared with pre-pandemic levels [2]. A few studies have examined resilience and its impact on mental health of nurses in the developing countries such as Philippine [3], Iran [16], and Turkey [17]; however, no studies of HCWs’ resilience have been conducted in Southeast Asia, including Nepal, Vietnam, and Taiwan.

We observed a statistically significantly higher resilience score for Vietnam and Taiwan, as compared with Hong Kong and Nepal with moderate mean—which might be due to higher satisfaction with organizational policy and least COVID-specific worries in Vietnam, as compared with least satisfaction of organizational policy and most COVID-specific worries in Nepal. Lowest resilience among HCWs in Nepal may be due to fear of social discrimination and neglect [18]. Furthermore, the survey period in Nepal took place from August to November 2021, right after the third peak of COVID-19 pandemic in which a surprising increase in the new cases and death rate were observed, especially in younger adults and people without comorbidities [19], likely reducing the HCWs’ resilience. By contrast, the survey period in Vietnam took place from July to November 2021 during the fourth wave of the pandemic, which was mainly related to the Delta variant when Vietnam’s authorities have had more time and experience to learn lessons and draw up strategies to better control and prevent the spread of COVID-19 [20]. Such strategies, including “Prepare a well-designed, sustainable preventive healthcare system from the grassroots level,” “Control the potential waves with a combination of contact tracing, isolation, and quarantine,” and “Priority the use of vaccine” [20], might increase resilience among HCWs in Vietnam. The survey period in Taiwan took place between December 2021 and July 2022, later than the other jurisdictions, during which the pandemic was dominated by the Omicron variant. The lesser severity of the symptoms presented by those infected by Omicron may also be associated with the relatively high resilience level in HCWs in Taiwan.

In addition, HK, Taiwan and Vietnam had experience of Severe Acute Respiratory Syndromes (SARS) cases among 30 jurisdictions between 1 November 2002 and 31 July 2003. This exposure could contribute to realizing the concepts of spiritual transformation [21] and/or emotional/posttraumatic growth [22], enabling individuals to look at things from a different and more adaptive perspective. Thus, their experience of the SARS pandemic could enhance post-traumatic growth in HCWs and organizations, protecting against the negative impact of the COVID pandemic more rapidly and effectively than transpired in Nepal, which did not experience the SARS outbreak. Promoting resilience in HCWs during the COVID-19 pandemic was reviewed and it was found that a relational leadership style, a supportive and safe work environment, and appropriate communication could support nurses’ resilience [23].

Our findings suggested that HCWs with older age, part-time work, higher education level, satisfaction with the workplace policy, organizational support, fewer COVID-specific worries, and fewer chronic health conditions are likely to have high levels of resilience. HCWs with older age may have more years of clinical experience, part-time workers tend to have less work stress, and those with higher education levels may have more ability to release their stress, all of which would result in higher resilience to tackle the challenges in healthcare services posed by the COVID pandemic [4, 24]. Satisfaction with the workplace infection control and prevention guideline, in terms of comprehensiveness, clarity, timeliness, transparency, or effectiveness, organizational supports of providing professional training/education and regular virus testing, less worried about the shortage of workplace protective equipment and the danger of infecting family members, were found to be positively associated with increased resilience in our study. Satisfaction with organizational policy could result in higher employee self-efficacy, enabling employees to better cope with negative situations, obstacles, and uncertainty, thus producing a more resilient atmosphere [25].

Our previous studies examined the association between satisfaction with workplace policy guidelines and COVID-specific worries with anxiety symptoms and posttraumatic growth among nurses [13, 26], and health status in the general population of Hong Kong [12]—but not on resilience. Among the four jurisdictions, HK, Taiwan and Vietnam, provided allowances other than duty allowance; this, in addition to the access to free COVID-19 vaccination to satisfy HCWs’ basic human needs. Salary and benefit packages were found to be one of crucial provisions of organization support that made employees feel trusted and valued in the workplace [27]. Our findings provide a novel insight that healthcare system policies and organizational support could constitute efficient measures/instruments to foster resilience of HCWs during the pandemic [28, 29]. Resilience is not solely an individual responsibility, but rather a mutual responsibility between the individual and the organization. Supportive strategies should target not only individual-level factors such as emotion regulation skills, but also social support which could be crucially enhanced across social and organizational settings [30]. Future international studies for improving resilience of HCWs should be provided at both the individual and organizational levels.

### Strengths and Limitations

This is the first study up to date to explore and compare the resilience level among HCWs under the high pressure of the COVID-19 pandemic in four different Southeast Asia jurisdictions and examined its associated factors. This study has found that generally the age, education level and experience of HCWs could affect their resilience during the pandemic. This multicenter study also strengths the idea that satisfying organizational policies and supportive work environments can enhance individual resilience for frontline HCWs [31]. Healthcare organizations therefore could have insights for considering the practical way to strengthen and implement related infection policies during the critical period. Current study also possesses several limitations. First, the cross-sectional study design could not uncover casual relationships between the identified associated factors and level of resilience among the HCWs. Future studies might examine the changes in workplace policy and organizational support to promote HCWs’ resilience using a longitudinal study design. Second, we employed a convenience sampling approach to recruit HCWs through the Association of Hong Kong Nursing Staff in Hong Kong; while in other jurisdictions, we used multiple social networks to invite the target populations to participate. HCWs recruited from different departments and relatively small samples collected in Vietnam, Nepal, and Taiwan might affect the interpretation of the results. Male HCWs and doctors were underrepresented in our samples. Thus, the findings of this survey could not be fully generalized to all doctors and nurses, as well as other healthcare professionals. Third, resilience scores on the CD-RISC tool might rely on the geographic location of the sample where the differences in cultures and healthcare systems prevents the findings from being more generalizable as well [32]. Therefore, our findings should be interpreted with caution.

### Conclusion and Interpretations

While facing the same virus, HCWs’ resilience was found to differ in Hong Kong, Nepal, Vietnam, and Taiwan during the COVID-19 pandemic. In general, participants with old age, part-time work, higher education level, more satisfaction with workplace policy, better organizational support, and fewer COVID-specific worries were associated with the higher resilience levels. A notable phenomenon was also observed for HCW’s resilience, in the relationship that emerged with the context of the pandemic that was experienced and organizational policy and support. The findings suggested the importance of designing and implementing resilience building programs at both individual and organizational levels. Implementing robust and suitable workplace policies and establishing supportive work environments, could help frontline HCWs to deal with the challenging situations they face. In addition, a benefits package could be a crucial motivator to recognize the HCWs’ efforts and trustworthiness in keeping the healthcare system afloat, and in turn potentially increasing their individual resilience and the stability of the wider organization they operate within.
